# How do the cognitive processes matter in the event-based preschoolers’ prospective memory?

**DOI:** 10.3389/fpsyg.2024.1279144

**Published:** 2024-04-18

**Authors:** Elżbieta Szpakiewicz, Małgorzata Stępień-Nycz

**Affiliations:** ^1^Institute of Psychology, Jagiellonian University, Krakow, Poland; ^2^Institute of Psychology, University of the National Education Commission, Krakow, Poland

**Keywords:** prospective memory, event-based, multiprocess theory, task focality, children, preschool-age, cognitive abilities, executive framework

## Abstract

**Background:**

Prospective memory (PM) is the ability to remember to perform an intended action at a specific future moment. The current study examined the impact of age, task focality, and cue salience on PM in children aged 2 to 6 years, based on the multiprocess theory of PM and the executive framework of PM development. Additionally, the study explored the relationship between various cognitive abilities and their association with PM performance.

**Methods:**

A total of 224 preschool-aged children, aged 2–6, engaged in event-based PM tasks with varying cognitive demands. The tasks were either focal or nonfocal, with salient or nonsalient cues. Additionally, individual differences in cognitive abilities were measured.

**Results:**

The results support previous indications that even very young children can successfully complete event-based PM tasks. The accuracy of PM display improved with age, especially between the ages of 3 and 4. Better performance was observed in focal PM tasks compared to nonfocal PM tasks. Additionally, preschoolers’ PM performance correlated with various cognitive abilities, including fluid intelligence, retrospective memory, inhibitory control, working memory, and language ability. These correlations varied depending on the child’s age and the task’s nature. For both focal and nonfocal PM tasks, cognitive abilities partially mediated the relationship between age and PM performance.

**Conclusion:**

In summary, this study comprehensively explores the specific roles played by age and fundamental cognitive abilities in event-based PM performance among preschool-aged children.

## 1 Introduction

The ability to remember to perform an intended activity at a specific point in the future is called prospective memory (PM; Einstein and McDaniel, 1990). PM plays a crucial role in a child’s personal autonomy and represents a key developmental milestone that facilitates children’s transition to independent individuals (Mahy et al., 2014a). Children encounter two types of PM tasks in their daily lives: event-based PM tasks and time-based PM tasks.

In event-based PM tasks, the ongoing task (OT; e.g., returning home) must be temporarily interrupted to carry out the intended action (e.g., asking a parent to purchase a toy) when the target cue emerges (e.g., a toy store). In contrast, in time-based PM tasks, the execution of the future intention (e.g., turning off a television) takes place after a predetermined period (e.g., 30 min). The cognitive demands of PM tasks vary. It is assumed that time-based PM tasks impose greater executive control demands on children compared to event-based PM tasks (Einstein and McDaniel, 1996). Due to the limited cognitive capacities of young children (Neumann et al., 2021) and their limited understanding of time (Brace et al., 2019), most studies involving preschoolers have primarily focused on event-based PM.

In recent years, there has been a significant increase in studies examining PM in children (see Mahy, 2022, for a review). However, there are still emerging themes in the early development of PM that require further in-depth research. One such theme is the validity of the predictions made by multiprocess theory (McDaniel and Einstein, 2000; Einstein and McDaniel, 2005; McDaniel and Einstein, 2007) in early childhood. The available evidence indicates that the multiprocess theory can indeed be effectively applied to preschooler research (cf. Mahy et al., 2014a). However, further research is required to determine the extent to which this theory can explain the mechanisms underlying children’s PM.

A multiprocess theory of PM is based on the assumption of the flexibility and complexity of the human cognitive system. Depending on the needs and requirements of the situation, PM can be mediated either by a automatic processes, or by controlled, strategic processes. While in some situations, the detection of a PM cue may require effortful monitoring of the environment, in other situations the PM cue will automatically trigger the intended action. McDaniel and Einstein (2000) proposed several factors that could determine whether event-based PM tasks invoke automatic or relatively effortful (i.e., controlled) retrieval processes. These factors include the importance of the PM task, the demands of the OT, the nature of the PM cue (salient vs. nonsalient), the characteristics of the PM task (more vs. less focal to the OT), and individual differences in cognitive resources. Event-based PM tasks can place varying demands on executive control processes, leading to different performance outcomes for children in various situations. The significance of PM task importance and OT demands has been extensively discussed in previous literature (e.g., Han et al., 2017). However, our understanding of the roles played by different types of PM cues and PM tasks remains limited. Therefore, the present study aims to explore the contributions of these elements to PM performance.

A key concept in understanding the multiprocess theory is focal processing. The multiprocess theory categorizes event-based PM tasks into two subtypes: focal and nonfocal tasks (McDaniel and Einstein, 2000). This categorization is based on the extent of procedural overlap between performing the OT and detecting the PM cue, which signals the need to perform the PM task. In a focal event-based PM task (henceforth referred to as focal PM tasks or focal task), there is high overlap between performing the OT and identifying the PM cue. For instance, if a child’s OT involves naming objects depicted on cards, a focal PM task would be to refrain from naming the object and instead place the card in a designated box whenever it shows an apple. Therefore, in this scenario, detection of the target cue can rely more strongly on automatic processes.

On the other hand, in nonfocal event-based PM tasks (henceforth referred to as nonfocal PM tasks or nonfocal tasks), there is a lower overlap between the OT and the PM tasks. For example, successfully detecting the PM cue in addition to naming the objects requires checking whether the object is a fruit. In this case, children must continuously strategically monitor for the appearance of the PM cue. This means that detecting the target cue depends on strategic processes and a broader range of cognitive abilities must be engaged for accurate PM task performance. Therefore, the potential challenges associated with maintaining a prospective intention while conducting an OT appear to be greater for a nonfocal PM task (cf. Cejudo et al., 2019). Current evidence suggests that children are more successful in performing focal tasks at an earlier stage of life compared to nonfocal tasks (however, see Kelly et al., 2023, for contrary findings). Further research is warranted to fully understand this distinction.

In addition to task focality, cue salience is also relevant to PM performance (McDaniel and Einstein, 2000). Cue salience is defined as the prominence of cues that signal an intended action (van Benthem et al., 2015). Some authors have suggested that salient or unusual cues may automatically and involuntarily trigger the intended action (Fuke and Mahy, 2022), thus potentially enhancing PM performance (Zhang et al., 2021). The multiprocess theory suggests that salient cues require fewer cognitive resources than nonsalient cues because they engage automatic processes, which refers to spontaneous non-attentional retrieval. Therefore, enhancing the salience of PM cues benefits PM performance in children, who have limited cognitive abilities (Mahy et al., 2014a).

It can be argued that the controlled processes, which are involved in PM, constitute the primary domain of executive functions (EF) (Mahy, 2022). Therefore, the executive framework of PM development (Mahy et al., 2014a) could be considered as an explanation for the mechanisms driving age-related changes in PM performance during childhood. The framework suggests that both efficient retrospective memory (RM) and strong EF, including working memory (WM), inhibitory control, switching, planning, and monitoring, are necessary to support PM. Some argue that RM and EF are crucial in the development of young children’s PM (see Mahy et al., 2014a).

RM is important for remembering the prospective intention, while WM plays a significant role in bringing this intention into the focus of attention. Inhibitory control and switching are responsible for detecting the PM cue, inhibiting the OT to perform the intended action, and switching between the PM task and the OT. Planning and monitoring are essential for both formulating intentions and supervising their execution, as well as detecting the PM cues (Hicks et al., 2000). According to the executive framework, children may need to rely on controlled executive processes for prospective memory tasks that are challenging for them. As cognitive abilities are still developing during early childhood (e.g., Diamond, 2002; Zelazo et al., 2003; Moriguchi, 2014; Anderson, 2022), age-related improvements in PM performance should be noticeable, particularly in tasks that require demanding controlled processes, such as nonfocal tasks with nonsalient cues. In this context, the executive framework of PM development aligns with the multiprocess theory (Mahy, 2021).

Some authors suggest that executive functioning and other cognitive abilities have varying impacts on different types of PM tasks (see Zuber et al., 2019, for an overview of studies examining the link between executive functioning and PM performance). A connection between overall cognitive functioning and PM performance is generally agreed upon by most authors. However, there is a lack of consensus on how they are associated with different types of PM tasks. It is still uncertain whether there are clear links between cognitive abilities and the performance of focal/nonfocal PM tasks with different levels of cue salience. The current study is the first to investigate various core cognitive abilities responsible for conscious control of thought and action, as well as four types of PM tasks with different resource demands. These include focal task with salient cue, focal task with nonsalient cue, nonfocal task with salient cue, and nonfocal task with nonsalient cue. The study was conducted on a group of preschool children spanning the entire age range.

The literature has established that the first successful PM performance can be observed in 2-year-olds (Somerville et al., 1983; Kliegel and Jäger, 2007; Ślusarczyk et al., 2018). Therefore, we included this age group in the current study to ensure that these children can execute an intended action in the future, even when there is no motivational aspect and the PM task is less or more demanding. As both PM and certain cognitive abilities, (such as EF) show significant and similar improvements during the preschool years (e.g., Guajardo and Best, 2000; Carlson and Moses, 2001; Kliegel and Jäger, 2007; Mahy and Moses, 2011), we chose to focus on 2- to 6-year-old children in this study to cover this period of rapid development.

### 1.1 The current study

Understanding the early development of PM and the role of cognitive abilities in preschoolers’ PM performance is crucial due to PM’s significance in children’s daily functioning. Improved knowledge of children’s PM could lead to better opportunities for supporting children’s autonomy and independence.

The aim of this study was to test the predictions of both the multiprocess theory and the executive framework of PM development by investigating whether cognitive abilities indeed predict children’s performance across diverse PM tasks during the preschool years (i.e., in 2- to 6-year-old children). To achieve this, we manipulated task focality (within participants) and cue salience (between participants) within a PM paradigm involving children aged 2 to 6. This allowed us to evaluate how different levels of executive demand affect the retrieval process in PM. According to the multiprocess theory, we predicted that the PM cue would automatically trigger the intended action in the focal tasks and the tasks with a salient cue, without requiring any specific cognitive abilities. Therefore, we expected good performance in these tasks, even in young children.

To measure the attentional demands of PM tasks, Smith (2003) developed “cost paradigm.” The paradigm suggests that adding an attentional PM task to the OT would decrease reaction time on the OT. However, according to multiprocess theory, this cost should be visible particularly in focal tasks. As target monitoring is a resource-demanding process, it can be particularly challenging for children (Bayen et al., 2019). In our study, we analyzed children’s monitoring during the OT in both focal and nonfocal tasks. Monitoring in PM is typically analyzed by comparing reaction times in OTs between different PM and control (no PM task) conditions, with monitoring costs measured as slower reaction times in PM conditions (e.g., McDaniel et al., 2011; Chi et al., 2014 for adults studies; Cejudo et al., 2019 for children study). Cejudo et al. (2019) conducted a study on children’s performance in a control condition, and compared it with performance in two PM conditions that varied in cue focality. The results showed that nonfocal PM cues compromise attentional control in children, as evidenced by greater reaction times in the nonfocal conditions. This finding is consistent with the multiprocess theory. In our research, we employed a distinct method for monitoring analysis, considering the reaction time before and after the occurrence of the PM cue. Consistent with the multiprocess theory, we hypothesized that tasks with high cognitive and attentional demands, requiring monitoring during the OT, would have longer reaction times before the PM cue than tasks with lower cognitive and attentional demands.

Additionally, we assessed individual differences in various cognitive abilities, including fluid intelligence, WM, inhibitory control, RM, and language abilities. We examined their relationship with PM performance and investigated whether these cognitive abilities mediated age effects on PM. Furthermore, we anticipated that PM tasks that do not necessitate additional cognitive resources to monitor the environment (i.e., the focal tasks, the tasks with salient cue) would not correlate with the performance of tasks measuring cognitive abilities. In these cases, the PM cue will automatically trigger the intended action, and no additional cognitive abilities are required for good PM performance.

Our hypotheses were as follows:

1. The performance in a focal PM task with a salient cue in the youngest children (2-year-olds) will be above chance level. This type of task does not require additional cognitive abilities for controlled monitoring of the environment for a target event. Therefore, even children who are still developing their cognitive abilities should be able to perform it.
2. Children will perform better in a PM task with a salient cue compared to a nonsalient cue. This effect will be particularly prominent among younger children.
3. In a focal PM task, children’s performance is expected to be better than in a nonfocal PM task. This difference will be more noticeable in younger children due to their limited cognitive abilities.
4. In tasks with high cognitive demand (i.e., nonfocal tasks), reaction times in the OT will be longer compared to tasks with lower cognitive demands.
5. Older children will demonstrate better PM task performance compared to younger children. This is based on previous studies highlighting developmental differences in PM within this age range, as well as the cognitive advantages specific to this age range. The difference in task performance is expected to be particularly noticeable between 3- and 5-year-old children.
6. Performance in the nonfocal PM task will correlate with cognitive abilities.
7. Performance in the PM task with a nonsalient cue will correlate with cognitive abilities.
8. The relationship between age and performance in PM tasks will be mediated by cognitive abilities.

## 2 Materials and methods

### 2.1 Participants

Two hundred and fifty-one children between the ages of 2 and 6 years (28–82 months) participated in the study. The children were divided into five age groups: 2-, 3-, 4-, 5-, and 6-year-olds. A total of 27 children who could not accurately reproduce the PM instruction, even after the most specific prompt (see the Procedure section), were excluded from the final sample and subsequent analyses (thirteen 2-year-olds, ten 3-year-olds, two 4-year-olds and two 6-year-olds). Therefore, the final sample consisted of 224 children (M_age_ = 56.01 months, SD = 15.52), including 117 girls (52%; there was no significant age difference between the two gender groups). None of the participants had a reported history of neuropsychopathology or psychopathology, as assessed by their kindergarten teachers, nor did any have intellectual deficits. Children were recruited through their kindergartens, and informed written parental consent was obtained for all participants. All children lived in southern Poland, primarily (80%) in urban areas. Upon completion of the entire study, participants were given small gifts in the form of a mascot of an animal with horns (see the Procedure section). Table 1 presents the final sample’s number of children, gender distribution, and mean age per age group.

**Table 1:** Number of participants, gender distribution, and mean age per age group of the final sample.

	*N*	Girls	Age range	M_age_ (SD)
Overall	224	117	28–82	56.01 (15.52)
2-years	36	16	28–35	32.31 (1.94)
3-years	39	25	37–47	42.13 (3.71)
4-years	47	22	48–59	54.32 (3.67)
5-years	50	27	60–71	65.62 (3.60)
6-years	52	27	72–82	75.13 (2.48)

N, number of participants; Age, age in months.

### 2.2 Design

We used a 5 × 2 × 2 mixed factorial design with age (2, 3, 4, 5, 6 years) and *cue salience* (nonsalient vs. salient) as between-subjects factors, and *task focality* (focal vs. nonfocal) as within-subjects factor. To prevent any potential negative effects of within-subjects manipulation, the focal and nonfocal tasks were separated by a one-week interval, with the order of the conditions randomized. The prospective tasks used in this experiment were classified as focal or nonfocal based on their description proposed by McDaniel and Einstein (2000). The study employed card-sorting tasks (e.g., Kvavilashvili et al., 2001; Mahy and Moses, 2011; Mahy et al., 2014b) following the standard PM procedure.

### 2.3 Measures

#### 2.3.1 Prospective memory

For the PM task, children played a card naming game adapted from Kvavilashvili et al. (2001), which we referred to as the “Marcel the Mole task” in our study. In the OT, children were instructed to name four stacks of cards (14.8 cm x 10.5 cm) featuring different animals. The animal images on the cards were selected through a pilot study to ensure that all the animals were familiar to children aged 2 to 6. All animal drawings were created by an artist and presented in a child-friendly style. To standardize the card sets, cards with similar appearances, such as body color, and belonging to the same category were included in each stack. For example, a seal was included in the first stack, a whale in the second stack, a shark in the third stack, and a dolphin in the fourth stack. The sequence of presentations for the four stacks and the cards within each stack was consistent for all children. However, the number of presented cards varied depending on the child’s age. The card count in each stack was adjusted based on the pilot study that determined how many animals a child in a specific age group could name within 1 minute. For 2-year-olds, 11 cards were presented, for 3-year-olds, 15 cards were presented, for 4-year-olds and 5-year-olds, 23 cards were presented, and for 6-year-olds, 27 cards were presented.

The PM task involved remembering to refrain from naming the card and instead placing it in the box whenever the image depicted a goat (focal condition) or an animal with horns (nonfocal condition). Each stack contained one of four different images of goats or animals with horns, with the target picture positioned differently in each stack. To ensure consistent timing of card presentation, the position of each PM trial varied across age groups due to the varying number of cards in the stacks according to the child’s age (see Table 2).

**Table 2:** The positions of target cards in each age group.

	2-year-olds	3-year-olds	4-year-olds	5-year-olds	6-year-olds
1. stack	5	6	9	9	11
2. stack	9	12	18	18	21
3. stack	7	9	14	14	16
4. stack	3	3	5	5	6
Total number of cards in each stack	11	15	23	23	27

In the nonsalient condition, all cards (including target cards) were white. In the salient condition, however, the target cards were yellow while the others remained white.

#### 2.3.2 Retrospective memory

##### 2.3.2.1 Card recognition test

Directly following the completion of Marcel the Mole task (see below in the Procedure section), children were given a recognition test using cards from the fourth stack (see Mahy and Moses, 2011; for the original procedure). The test consisted of 20 non-target cards, half of which were previously shown to the children. They were then asked to indicate whether they had seen these cards during the game with Marcel the Mole. The novel cards belonged to the same general categories as the previously shown cards and had similar appearances (e.g., lizard vs. chameleon, butterfly vs. dragonfly, parrot vs. woodpecker). The accuracy of the children’s recognition was evaluated out of a total of 20 points.

##### 2.3.2.2 Auditory memory task

The Auditory Memory Task from the Intelligence and Development Scales for Pre-school Children (Grob et al., 2015) was administered in its Polish version. The task required the child to recall a story that they had heard 30 min earlier. If the child was unable to recall the story spontaneously, additional questions about the details were presented. Points were assigned according to the following criteria: each instance of spontaneously recalling a significant story detail is worth 2 points, while recall prompted by supplementary questions is worth 1 point. The scoring range is from 0 to 20. The reliability of the task is high: rtt = 0.73 in one-month interval, and split-half parallel reliability coefficient calculated with the Spearman-Brown formula = 0.83 (Fecenec et al., 2015).

As the tasks measuring RM were correlated (*r* = 0.52, *p* < 0.001), the mean value of the *Card Recognition Test* and the *Auditory Memory Task* was used to calculate the aggregated measure of RM.

#### 2.3.3 Fluid intelligence

The Raven’s Colored Progressive Matrices test (Szustrowa and Jaworowska, 2003) was administered following the standard Raven’s procedure (Raven et al., 1995). Children were presented with a set of six choices and asked to select the missing element. Each correct answer was awarded one point, and the overall score was the sum of the correct responses, with the highest possible score being 36. The reliability of the task is high: rtt = 0.90 (Raven et al., 1995).

#### 2.3.4 Language ability

The Picture Vocabulary Test - Comprehension (Haman et al., 2012) was used to evaluate the language skills of Polish-speaking children aged 2; 0–6; 11 years. The test assesses the comprehension of nouns, verbs, and adjectives using four colored pictures for each item: one target image and three distractor images. The score is based on the number of correct responses, with a range of 0 to 86. The reliability of the task is high: split-half parallel reliability coefficient calculated with the Spearman-Brown formula exceeds 0.90 (Haman et al., 2012).

#### 2.3.5 Inhibitory control

##### 2.3.5.1 Day/night task

To perform the Day/night Stroop-like task (Gerstadt et al., 1994; review: Montgomery and Koeltzow, 2010), children are required to say the opposite of what the stimulus cards represent. The task involves a total of 20 cards, consisting of 4 trial cards and 16 test cards with 8 sun and 8 moon cards. Half of the cards depict a yellow sun on a white background, while the other half depict a yellow moon and silver stars on a black background. The task was to say “night” when the sun was shown on the card and say “day” whenever the card showed the moon with stars. Once the child had accurately responded in two consecutive practice trials, the standardized testing phase began. The cards were presented in the pseudorandom order of suns and moons. The accuracy of the responses was recorded and scored out of 16.

##### 2.3.5.2 Bear/dragon task

The Bear/Dragon task (Reed et al., 1984) was administered as a well-known “go vs. no-go” task, also known as the Simon Says task. A friendly bear puppet and naughty dragon puppet were introduced to a child. The child was then instructed to obey the bear’s commands (e.g., “touch your head”), while refraining from following the dragon’s instructions. After the practice phase, a set of 10 test trials followed, wherein the bear and dragon commands were presented in a semi-alternating order. The scoring range spans from 0 to 5, exclusively accounting for no-go responses.

##### 2.3.5.3 Visual Simon task

The Visual Simon Task, which is a modified version of the Spatial Conflict task (Gerardi-Caulton, 2000), involved presenting children with two types of visual stimuli: yellow and blue fish, which children were supposed to “feed.” Children were instructed to respond to these stimuli by making a rightward response to a yellow fish and a leftward response to a blue fish, executed by pressing the corresponding button on the keyboard (either yellow or blue). These stimuli were displayed individually on either the left or right side of the screen. The location of the display, where the stimuli were shown, influenced the way children responded. It led to either matching responses (congruent trials) when the side of the correct button press aligned with the fish’s color or non-matching responses (incongruent trials) when it did not align. The scoring for this task ranged from 0 to 13, with only the incongruent trials being considered.

As all above tasks correlated significantly (*r*s ranged from 0.36 to 0.57, all *p*s < 0.001), the aggregated measure of inhibitory control was calculated as the mean value of the *Day/night task*, the *Bear/Dragon task*, and the *Visual Simon Task*.

#### 2.3.6 Working memory

##### 2.3.6.1 Nonverbal working memory

Nonverbal WM was assessed using a modified child-friendly computer version of the Forward and Backward Corsi Block-Tapping Tasks (Corsi, 1972). In the forward task, children were asked to tap a sequence of illuminated blocks depicting objects such as a house or a flower, in the same order as displayed on the screen. The length of the sequence increased from two to six pictures. Participants began with two sequences, each consisting of two blocks. If they accurately replicated one or both of these sequences, they advanced to more difficult sequences with an additional block. The task ended when the participant failed two consecutive trials. The backward task was administered after completing the forward task. The standard scoring procedure proposed by Corsi was used to rate each trial, with a score of 0 or 1 being multiplied by the number of blocks that should have been pressed. The Nonverbal Working Memory final score ranged from 0 to 80, with 0–40 for the Forward Corsi Block-Tapping Task and 0–40 for the Backward Corsi Block-Tapping Task.

##### 2.3.6.2 Verbal working memory

Verbal WM was assessed using the Forward and Backward Digit Span Task from the Wechsler Intelligence Scale for Children - Revised (Matczak et al., 2008). In the Forward Digit Span Task, participants repeated a sequence of numbers in the same order as spoken by the experimenter. An additional number was incrementally introduced after successfully completing one or both of two trials per length. The task ended when children failed two consecutive trials. For the Backward Digit Span Task, participants repeated the numbers in reverse order. This makes the procedure comparable to the Forward and Backward Corsi Block-Tapping Task (described above). The score for the Forward Digit Span Task was calculated by summing the number of accurately repeated forward digit strings, with a maximum of 14 points. Similarly, the score for the Backward Digit Span Task was based on accurately repeated sequences of digits in reverse order, with a maximum of 14 points. The reliability of the task is high: rtt = 0.70 (Matczak et al., 2008).

As nonverbal and verbal memory tasks were significantly correlated (*r* = 0.49, *p* < 0.001), the aggregated measure of WM was calculated as the mean value of Nonverbal Working Memory and Verbal Working Memory.

#### 2.3.7 Switching

The Children Card Sort (Jabłoński et al., 2018) is a method that is standardized for Polish-speaking children and is based on the Dimensional Change Card Sorting (Zelazo, 2006). In the first two stages, children were asked to sort a series of bivalent test cards (depicting a house or a cat; red or blue), initially based on their color (the color phase), and then based on their shape (the shape phase). In the final stage, the researcher introduced a new sorting rule based on the presence of a border on the cards. If a card had a border, it signaled sorting by color, while borderless cards indicated sorting by shape. The researcher presented subsequent cards to the child, recalling the sorting rule (color vs. shape) for each card. This required the child to shift from one sorting strategy to another. Only children who accurately sorted a minimum of five cards in both the color and shape phases were assessed in the border phase (see Jabłoński et al., 2012, for the same procedure). Within our study, 41 children did not meet this criterion: 18 two-year-olds, 12 three-year-olds, 9 four-year-olds, and 2 six-year-olds. The final result represented the total count of accurately organized cards within the border phase, with a range of 0–12. Reliability of this task, calculated with the use of Guttman’s lambda-6, is 0.73–0.95 (Jabłoński et al., 2018).

#### 2.3.8 Attention

The Selective Attention subtest from the Intelligence and Development Scales for Pre-school Children (Grob et al., 2015) was used in its Polish version. The task required children to sort cards featuring ducks based on the color of their beaks (yellow vs. white). Although some cards also depicted a yellow sun (as a distraction), children were instructed to disregard it. The final score is determined by the number of cards correctly sorted within a 90-s time frame, with possible scores ranging from 0 to 72.

#### 2.3.9 Planning

A computerized adaptation of the Tower of London task (Krikorian et al., 1994) was used in this study. The task required the child to manipulate balls on pegs using a computer mouse to achieve a target configuration displayed on the upper screen. The child could complete the task independently or with experimenter assistance if needed. The task was to complete it within a predetermined number of moves, following five rules: (1) move only one ball at a time, (2) do not pick up more than one ball at once, (3) place balls only on the three pegs, (4) do not move a ball in a lower-row if another ball is above it, and (5) follow the peg-specific capacities (three on the left, two on the middle, and one on the right). The computerized version of the task made it impossible to violate rules, such as moving two balls at once or placing them outside of the pegs. This feature relieved children from the need to memorize the rules. The task consisted of one practice and 12 test problems. Scores were based on the number of correctly solved trials within the allowed moves (range: 0–12).

### 2.4 Procedure

The children were individually tested in a small, distraction-free room over three sessions, each separated by a two-week delay. Each session lasted approximately 60 min. During the first session, a warm-up game was played with the experimenter to familiarize the child with them. Then, the child was introduced to a stuffed animal named Marcel the Mole. Marcel had a birthday party, but due to his poor daytime vision, he was unable to identify who attended. For the OT, participants were instructed to help Marcel by verbally labeling depicted animals. Children began with four practice trails, such as a pig. They were also informed that Marcel would be happy if they drew some birthday cards for him throughout the session. The experimenter then introduced the PM task. The children were informed that Marcel had a fear of the goats/animals with horns, so they were asked not to name them and to hide any cards with a goat/ an animal with horns on them in a box located behind them. To ensure appropriate comprehension and memorization of the instructions, the child was asked to repeat the PM task instructions in their own words. Whenever there was any misunderstanding, the task instructions were repeated. All children were able to repeat the instructions correctly after at most one correction. When there were no questions or misunderstandings, a child had to draw a first picture for Marcel and then name the pictures in the first stack of cards. This procedure was repeated until all four stacks of cards were named and four pictures were drawn. The dependent measures were the number of correct responses and the reaction time measured using a stopwatch. Specifically, the reaction time was defined as the time between showing the card and the child naming it. To ensure that children’s errors are truly prospective (i.e., forgetting to carry out the intention at the correct time), in those cases where the child did not perform the PM task spontaneously, they were asked some questions about the task that they had to perform. First, the experimenter gave them a general prompt: “Was there something you were supposed to remember?” If the child still did not perform the PM task, this general prompt was followed by a more specific prompt: “You were naming the animals and drawing the pictures. Do you remember, what else did you have to do?” If the child still failed to perform the prospective action, a more specific prompt was applied: “Do you remember, was there something you were supposed to do when you saw a particular picture on the card?” If the child still did not perform the PM task, they received the most specific prompt: “Do you remember, was there something you were supposed to do when you saw a goat/an animal with horns”?

Following the PM task (focal or nonfocal; depending on the randomly selected order of performing PM tasks), a *Card recognition test* was administered. Then, *Raven’s Colored Progressive Matrices* and *Picture Vocabulary Test* were applied.

The second session began with the second PM task. Following this, tasks measuring inhibitory control and WM were administered. During the third session children performed *Children Card Sort, Selective Attention, Auditory memory task*, and *Tower of London*. At the end of the last session, participants received small gifts in the form of mascots of an animal with horns: goats, bulls, cows, reindeer, rams, and others. This procedure was done to ensure that children would not develop a fear of horned animals after participating in the study.

### 2.5 Statistical analysis

For PM tasks, there was no missing data. However, there was single missing data for the cognitive tasks, ranging from one case (for language ability) to 32 cases (for switching). Missing data were observed mainly in the groups of 2-year-olds (from 0% for language ability and attention to 41.7% for planning, 36.1% for switching and WM, and 30.6% for inhibitory control) and 3-year-olds (from 0% for attention and RM to 23.1% in switching and 12.8% in WM). Missing data in switching tasks were observed also in 4-year-olds (19.1%). In the remaining groups missing data did not exceed 2%. In each age group, data were missing completely at random (Little’s MCAR test was non-significant, all *p*s > 0.10). As missing data related to the whole tasks and not the single items in the tasks, we decided to pair-wise delete cases with missing data instead of imputing them.

For all analyses, an alpha level of 0.05 was applied. In the first step, descriptive statistics were calculated and preliminary ANOVA was performed to check for gender differences. As there were no differences between boys and girls in any measure of cognitive abilities and PM (all *p*s > 0.05), the following analyses did not take gender as a variable into account.

To assess the performance level of the youngest, 2-year-old children in PM tasks, we conducted one-sample t-tests to compare their performance to zero. We then performed mixed ANOVA with age group and cue salience as between-individual factors, and task focality as a within-individual factor. Cue salience has been designed as an between-individual factor to reduce the number of tasks required of the child. This approach was adopted due to the potential fatigue experienced by the child when faced with an excessive workload, particularly relevant in the context of younger children. The dependent variable was the number of correct responses. We used contrast analysis to compare the results of children in different age groups.

Finally, correlational analysis was performed to assess relations between PM performance and children’s cognitive abilities, followed by regression analyses. All analyses were performed using PS Imago PRO 9 software and PROCESS procedure for SPSS v. 4.2 (Hayes, 2022).

## 3 Results

### 3.1 Descriptive statistics

Table 3 presents the descriptive statistics for the PM tasks in different age groups, considering task focality and cue salience. Descriptive statistics calculated for cognitive abilities are presented in Appendix Table 1A.

**Table 3:** Descriptive statistics for the PM tasks.

PM task	Descriptive statistics (*M*; SD; range in parentheses)					
	2-year-olds	3-year-olds	4-year-olds	5-year-olds	6-year-olds	total
Focal task, salient cue	1.68; 2.00 (0–7)	3.05; 2.89 (0–8)	4.43; 3.60 (0–8)	7.04; 1.95 (2–8)	6.25; 2.34 (0–8)	4.72; 3.23 (0–8)
Focal task, nonsalient cue	2.41; 2.90 (0–8)	2.71; 2.76 (0–8)	4.96; 3.09 (0–8)	6.40; 2.35 (1–8)	5.92; 2.84 (0–8)	4.75; 3.16 (0–8)
Total score, focal task	2.03; 2.46 (0–8)	2.90; 2.80 (0–8)	4.70; 3.32 (0–8)	6.72; 2.16 (1–8)	6.10; 2.56 (0–8)	4.73; 3.19 (0–8)
Nonfocal task, salient cue	1.53; 2.07 (0–8)	3.59; 2.22 (0–8)	4.09; 3.00 (0–8)	5.92; 2.97 (0–8)	5.39; 3.14 (0–8)	4.28; 3.10 (0–8)
Nonfocal task, nonsalient cue	1.41; 2.21 (0–7)	2.59; 2.83 (0–8)	3.67; 2.65 (0–8)	4.72; 2.42 (0–8)	5.79; 2.57 (0–8)	3.86; 2.91 (0–8)
Total score, nonfocal task	1.47; 2.10 (0–8)	3.15; 2.52 (0–8)	3.87; 2.80 (0–8)	5.32; 2.75 (0–8)	5.58; 2.87 (0–8)	4.08; 3.01 (0–8)

### 3.2 PM in 2-year-olds

The percentage of 2-year-olds who scored at least one point in PM tasks with a salient cue was 58.8% in the focal task and 52.6% in the nonfocal task. A one-sample *t*-test revealed that the PM performance of 2-year-olds was significantly above zero, *t*(18) = 3.96, *p* < 0.001, Cohen’s *d* = 0.91. Additionally, the PM performance for focal and nonfocal tasks separately was greater than zero, *t*(18) = 3.67, *p <* 0.001, Cohen’s *d* = 0.84 and *t*(18) = 3.22, *p* = 0.002, Cohen’s *d* = 0.74, respectively. Similar results were obtained regardless of the salience of the cue (for focal tasks *t*(35) = 4.96, *p* < 0.001, Cohen’s *d* = 0.83, for nonfocal tasks *t*(35) = 4.20, *p* < 0.001, Cohen’s *d* = 0.70, respectively).

### 3.3 Cue salience and task focality

A mixed ANOVA revealed no main effect of cue salience, *F*_(1,214)_ = 0.59, *p* = 0.442, η2p = 0.003, or cue salience x age group interaction, *F*_(4,214)_ = 0.58, *p* = 0.681, η2p = 0.01. The main effect of task focality was significant, *F*_(1,214)_ = 10.87, *p* = 0.001, η^2^_p_ = 0.05, with focal tasks being easier than nonfocal tasks (see Table 3 for the means). There was no significant interaction effect of task focality x cue salience, *F*_(1,214)_ = 1.49, *p* = 0.224, η^2^_p_ = 0.007, or task focality x age, *F*_(4,214)_ = 1.96, *p* = 0.102, η^2^_p_ = 0.04. The detailed results of the MANOVA analysis are presented Appendix Table 2A.

### 3.4 The ongoing task

*Assessing children’s accuracy in naming the animals depicted on the cards was deemed unnecessary*. The animals presented on the cards were selected based on a pilot study to ensure familiarity to children aged 2–6, resulting in a near-ceiling level of OT performance across all age groups. The OT response time (before PM cue occurred) was longer for nonfocal tasks (2.04 s.) compared to focal tasks (1.97 s.), *F*_(1,141)_ = 4.04, *p* = 0.046, η^2^_p_ = 0.03, but this effect was very small. There was no significant interaction between the OT response time and cue salience, *F*_(1,141)_ = 0.22, *p* = 0.638, η^2^_p_ = 0.002, nor between the OT response time and age group, *F*_(4,141)_ = 0.93, *p* = 0.450, η^2^_p_ = 0.03.

### 3.5 Age and PM performance

**Figure 1:**
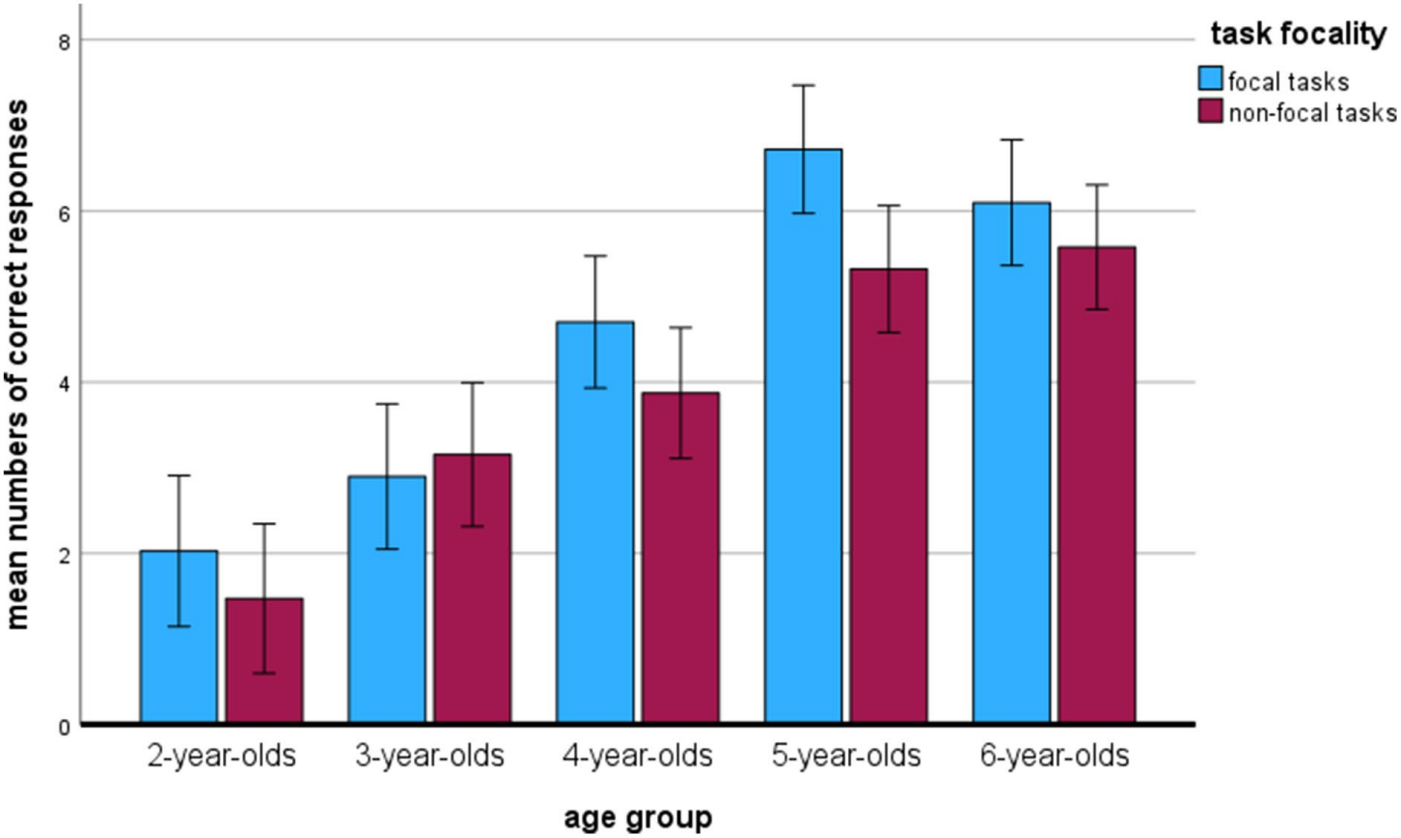
Prospective memory performance as a function of age (maximum = 8). Error bars represent the 95% confidence intervals for the means.

In the MANOVA analysis (see Table 2A), the main effect of age was observed, *F*_(4,214)_ = 26.72, *p* < 0.001, η^2^_p_ = 0.33. The repeated contrast analysis indicated significant differences between all age groups (*p*s < 0.05), except for the two oldest groups of 5- and 6-year-olds (*p* = 0.690; see Table 3 for the means). For the focal tasks, the 2-year-olds did not differ significantly from 3-year-olds (*p* = 1.00), whereas 6-year-olds did not differ from 4-year-olds (*p* = 0.116) and 5-year-olds (*p* = 1.00). For the nonfocal tasks, there was no significant difference between the 2-year-olds and 3-year-olds (*p* = 0.094). 5-year-olds did not differ from 4-year-olds (*p* = 0.082) and 6-year-olds (*p* = 1.00). Figure 1 shows the mean number of successful PM responses for both focal and nonfocal tasks as a function of age.

**Table 4:** Zero-order and partial correlations with age controlled for (in parentheses) for PM performance and cognitive abilities.

Task	Intelligence	Language ability	Attention	Retrospective memory	Planning	Switching	Inhibitory control	Working memory
Focal task, low salience	0.38*** (0.06)	0.55*** (0.27**)	0.41*** (0.04)	0.46*** (0.16)	0.33*** (−0.02)	0.15 (−0.004)	0.46*** (0.13)	0.43*** (0.12)
Focal task, high salience	0.54*** (0.27**)	0.57*** (0.22*)	0.41*** (−0.02)	0.61*** (0.33***)	0.48*** (0.16)	0.21* (0.03)	0.50*** (0.15)	0.49*** (0.16*)
Focal task	0.46*** (0.16**)	0.56*** (0.24***)	0.41*** (0.02)	0.54*** (0.24***)	0.40*** (0.05)	0.18** (0.01)	0.48*** (0.13*)	0.46*** (0.14*)
Nonfocal task, low salience	0.48*** (0.21*)	0.54*** (0.25**)	0.44*** (0.08)	0.45*** (0.13)	0.38*** (0.05)	0.07 (−0.11)	0.46*** (0.10)	0.46*** (0.16)
Nonfocal task, high salience	0.51*** (0.32***)	0.49*** (0.23**)	0.42*** (0.14)	0.48*** (0.23**)	0.42*** (0.17*)	0.36*** (0.25**)	0.45*** (0.21*)	0.44*** (0.19*)
Nonfocal task	0.49*** (0.26***)	0.51*** (0.23***)	0.42*** (0.10)	0.46*** (0.17**)	0.39*** (0.10)	0.21** (0.07)	0.46*** (0.17**)	0.45*** (0.17**)
Low salience	0.48*** (0.15)	0.61*** (0.31***)	0.48*** (0.07)	0.51*** (0.17*)	0.40*** (0.02)	0.13 (−0.06)	0.52*** (0.14)	0.51*** (0.16)
High salience	0.59*** (0.34***)	0.59*** (0.26**)	0.46*** (0.07)	0.61*** (0.33***)	0.50*** (0.19*)	0.31*** (0.16*)	0.53*** (0.21*)	0.51*** (0.20*)
PM general	0.53*** (0.25***)	0.60*** (0.27***)	0.46*** (0.06)	0.56*** (0.24***)	0.44*** (0.09)	0.22** (0.05)	0.52*** (0.18**)	0.51*** (0.18**)

*p < 0.05; **p < 0.01; ***p < 0.001, one-tailed.

### 3.6 PM performance and cognitive abilities

*Z*ero-order and partial correlations, controlling for age, were conducted separately for the focal and nonfocal tasks, as well as for tasks with highly salient and nonsalient cues. The correlational analysis results are presented in Table 4.

Generally, controlling for age, correlations between PM and cognitive abilities remained significant for general intelligence, language ability, RM, WM and inhibitory control. However, these correlations were differentiated mainly regarding cue salience. Correlations with WM and inhibitory control were not significant for tasks with nonsalient cues when controlling for age, contrary to expectations.

As younger and older children differed not only in the level of PM performance, but also in the level of their cognitive abilities, we conducted separate correlational analyses for younger (2- and 3-year-old) and older (5- and 6-year-old) children. The results are presented in Table 5. Generally, the correlations were higher for younger children, particularly in the nonfocal tasks.

**Table 5:** Pearson correlations between PM performance and cognitive abilities for younger and older children.

PM tasks	Focal tasks		Nonfocal tasks		PM general	
Cognitive abilities	Younger children	Older children	Younger children	Older children	Younger children	Older children
Intelligence	0.24*	0.18*	0.39***	0.27**	0.36**	0.26**
Language ability	0.15	0.09	0.44***	*0.03*	0.33**	*0.07*
Attention	0.08	−0.06	0.34**	*0.05*	0.23*	*−0.001*
Retrospective memory	0.22*	0.24**	0.34**	*0.11*	0.32**	0.19*
Planning	0.03	−0.01	0.23*	0.15	0.14	0.09
Switching	−0.08	0.06	0.08	0.07	−0.001	0.08
Inhibitory control	0.26*	*0.12*	0.53***	*0.10*	0.42***	*0.12*
Working memory	0.14	0.14	0.33**	*0.14*	0.26*	0.16

*p* < 0.05; **p < 0.01; ***p < 0.001, one-tailed; with italics correlation coefficients significantly different between younger and older children are marked (calculated using Fisher’s z-Test; *p*s < 0.05).

Finally, we conducted regression analyses with forward selection for the focal and nonfocal tasks separately. To account for high collinearity among the cognitive predictors, *z* scores of independent variables, transformed into the orthogonal factor scores with the principal component analysis (PCA) were used in the models. Transforming the predictors into orthogonal factor scores through PCA helps mitigate issues associated with multicollinearity, such as inflated standard errors and unstable estimates of predictor effects. For the entire group of 2- to 6-year-olds, the significant predictors of PM performance in the focal tasks were age (*β* = 0.59, *p* < 0.001, Δ*R*^2^ = 0.29) and attention (*β* = −0.18, *p* = 0.007, Δ*R*^2^ = 0.03), explaining together 32% of the variance in the focal tasks performance (*F*_(2,169)_ = 38.95, *p* < 0.001). Age and general intelligence were found to be significant predictors of PM performance in the nonfocal tasks (*β* = 0.42, *p* < 0.001, Δ*R*^2^ = 0.22 and *β* = 0.16, *p* = 0.022, Δ*R*^2^ = 0.02, respectively), explaining together 25% of the variance in the nonfocal tasks performance (*F*_(2,169)_ = 27.89, *p* < 0.001).

When conducting regression analyses separately for the younger (2- and 3-year-old) and older (5- and 6-year-old) children, only age was found to be a significant predictor of PM performance in nonfocal tasks for younger children (*β* = 0.39, *p* = 0.020, Δ*R*^2^ = 0.15, *F*_(1,34)_ = 5.94, *p* = 0.020). None of the variables predicted PM performance in the focal tasks. In the older group, the performance of PM in the focal tasks was predicted by RM (*β* = 0.20, *p* = 0.050, Δ*R*^2^ = 0.04, *F*_(1,98)_ = 3.94, *p* = 0.050), whereas in the nonfocal tasks, it was predicted by general intelligence (*β* = 0.22, *p* = 0.025, Δ*R*^2^ = 0.05, *F*_(1,98)_ = 5.16, *p* = 0.025). For the group of 4-year-olds who scored between younger and older children in the PM tasks, age (*β* = 0.30, *p* = 0.049, ΔR^2^ = 0.15) and language ability (*β* = 0.42, *p* = 0.007, Δ*R*^2^ = 0.17) were significant predictors of PM performance in the focal tasks, explaining together 32% of the variance (*F*_(2,33)_ = 7.70, *p* = 0.002), in the nonfocal tasks the only significant predictor was language ability (*β* = 0.40, *p* = 0.017, Δ*R*^2^ = 0.16, *F*_(1,34)_ = 6.27, *p* = 0.017).

Considering cue salience, in the nonsalient cue condition significant predictor of the PM performance in focal tasks was only age (*β* = 0.49, *p* < 0.001, Δ*R*^2^ = 0.24, *F*_(1,84)_ = 260.84, *p* < 0.001), similarly as in nonfocal tasks (*β* = 0.51, *p* < 0.001, Δ*R*^2^ = 0.26, *F*_(1,84)_ = 28.81, *p* < 0.001). In the high salience condition, significant predictors of the PM performance in the focal tasks were age (*β* = 0.50, *p* < 0.001, Δ*R*^2^ = 0.33) and RM (*β* = 0.22, *p* < 0.020, Δ*R*^2^ = 0.04), explaining together 37% of the variance in PM (*F*_(2,83)_ = 24.36, *p* < 0.001), whereas in the nonfocal tasks age (*β* = 0.39, *p* < 0.001, Δ*R*^2^ = 0.21), intelligence (*β* = 0.21, *p* = 0.036, Δ*R*^2^ = 0.04), and switching (*β* = 0.19, *p* = 0.041, Δ*R*^2^ = 0.04) explaining together 28% of the variance in PM (*F*_(3,82)_ = 10.80, *p* < 0.001).

Finally, mediation analyses were conducted using the PROCESS macro, with PM performance as a dependent variable, age as predictor, and those cognitive abilities that significantly correlated with PM when controlling for age (see Table 4) as mediators. Orthogonal factor scores were used for cognitive abilities instead of raw scores. Age significantly predicted all cognitive abilities included as mediators, as well as outcome variables (all *p*s < 0.05).

For the nonfocal tasks, the complete model significantly predicted PM performance (*R*^2^ = 0.23, *F*_(6,165)_ = 8.26, *p* < 0.001). The overall impact of age on PM performance was significant, *β* = 0.41, *p* < 0.001, *R*^2^ = 0.17. The direct effect of age was also significant, but smaller, *β* = 0.20, *p* = 0.030. The total indirect effects model was significant, *β* = 0.21 [0.06; 35]. The only significant predictor of performance was intelligence, *β* = 0.07 [0.02; 0.14], whereas the remaining mediators did not reach significance level (language ability *β* = 0.02 [−0.01; 0.06], inhibitory control *β* = 0.05 [−0.01; 0.11], RM *β* = 0.03 [−0.01; 0.07], WM *β* = 0.05 [−0.01; 0.10]).

The full model significantly predicted PM performance for the focal tasks (*R*^2^ = 0.31, *F*_(6,165)_ = 12.34, *p* < 0.001). The total effect of age on PM performance was significant, *β* = 0.49, *p* < 0.001, *R*^2^ = 0.24. The direct effect of age was also significant, but smaller, *β* = 0.24, *p* = 0.007. The total indirect effects model was significant, *β* = 0.24 [0.12; 36]. Three of the mediators reached significance level (inhibitory control *β* = 0.07 [0.01; 0.13], RM *β* = 0.05 [0.01; 0.09], and WM *β* = 0.06 [0.02; 0.12], whereas intelligence *β* = 0.04 [−0.01; 0.10] and language ability *β* = 0.03 [−0.002; 0.07] were non-significant). Therefore, for both focal and nonfocal tasks, cognitive abilities partially mediated the relationship between age and PM performance.

## 4 Discussion

The aim of the current study was to test the predictions of multiprocess theory (McDaniel and Einstein, 2000) and the executive framework of PM development (Mahy et al., 2014a). Specifically, we investigated whether there is a correlation between children’s PM performance and their cognitive abilities, such as WM, RM, and general intelligence, and whether these correlations change with age or the type of task.

Our first research question was whether 2-year-olds can perform laboratory event-based PM tasks without external incentives. We hypothesized that due to their limited cognitive resources and less developed monitoring abilities, 2-year-olds’ performance in nonfocal PM tasks would be inferior to that in focal PM tasks, as well as in tasks with nonsalient cues compared to those with salient cues. The study results suggest that cue salience has no effect on PM performance, regardless of any group. Both focal and nonfocal PM tasks were performed above zero by 2-year-old children, indicating that even with limited cognitive functioning, they were able to perform the tasks. Although the task required the child to hide an image of an animal with horns, in accordance with the multiprocess theory (McDaniel and Einstein, 2000) necessitated cognitive processes’ involvement, the study’s context was intriguing and engaging enough for the child to successfully perform it. In this context, our findings align with previous research (e.g., Kliegel and Jäger, 2007; Ślusarczyk et al., 2018). To answer the research question of whether children as young as 2 years old can perform a nonfocal task with a nonsalient cue more precisely, modifications to the nonsalient condition are necessary. It is possible that the task was inadequately designed in our study, which will be revisited later in the Discussion. It is hypothesized that the combination of nonfocal and nonsalient conditions may hold significance, given the substantial cognitive load involved. Therefore, future investigations should incorporate this aspect.

As hypothesized, children performed better in a focal PM task than in a nonfocal PM task. These results support the multiprocess theory, which suggests that nonfocal tasks require more cognitive monitoring to detect the PM cue, making them more challenging for children (Einstein and McDaniel, 2005). Confirming these findings, our study showed that in nonfocal tasks, where children needed to invest more effort to capture the prospective cue, the average reaction time in the OT (before the occurrence of the prospective cue) was longer than the comparatively measured time in the focal tasks. This reflects the additional cognitive load borne by children. To more comprehensively assess the costs incurred by the child in the OT in future studies, it is advisable to include a control condition. This should be a single-task condition where children are asked only to perform OT. This methodology was employed in the study involving school children by Cejudo et al. (2019).

Based on the findings of the study conducted by Zhang et al. (2021), which highlighted the facilitation of automatic processes in PM through salient cues, we hypothesized that the less salient the PM task cue, the more controlled monitoring may be required, potentially leading to larger age effects. However, the results obtained do not provide support for this hypothesis. A potential explanation for the absence of the salience effect could be that the cue’s salience must specifically relate to the target cue. In our study, the card’s background was salient (yellow cards compared to all other white cards), while the target cue (horns) remained the same across all conditions. Further research is required to support the hypothesis that improving PM performance necessitates cue salience to be directly linked to the stimulus itself, rather than the background. It is possible that if the horns were a different color in the salient condition, the effect of cue saliency would be observed. For instance, Kliegel et al. (2013) conducted a study (experiment 2) to investigate the impact of perceptual cue salience on age-related differences in event-based PM performance among primary school-age participants. The results showed that presenting a salient cue led to better PM performance than a nonsalient cue. In this study, in the nonsalient condition, one flowerpot on each side of the road contained yellow flowers, which contrasted with the standard soft pink flowers. In contrast, in the salience condition several yellow flower pots were presented.

Based on the multiprocess theory of PM, it is expected that there will be no significant age differences in the focal task, as it does not require attentional resources for spontaneous retrieval processes. However, a significant age-related effect is anticipated in the nonfocal task due to the challenging nature of effortful monitoring which may be particularly difficult for younger children to maintain consistently throughout the task. As developmental studies have shown a similar trajectory of cognitive abilities, including for example EF, which seem to be essential for PM, and PM itself (e.g., Guajardo and Best, 2000; Carlson and Moses, 2001; Kliegel and Jäger, 2007; Mahy and Moses, 2011), we expected an improvement in nonfocal PM tasks performance during early childhood, specifically between ages 3 and 5. Our study showed a general effect of age on PM performance, but not a differential effect/interaction with focality. Future longitudinal studies should include children aged 3 (as 2-year-olds exhibit only initial manifestations of PM) to 5 (since PM abilities stabilize from the age of 5 onwards) to capture the emerging developmental changes in PM abilities.

Successful performance of demanding PM tasks requires the recruitment and coordination of several cognitive processes (Sheppard et al., 2018). Therefore, it was hypothesized that children with limited cognitive resources would find more demanding PM tasks challenging. Some of the correlations between cognitive abilities and PM lose their significance when age is controlled for, such as for example attention, planning, and switching. This result suggests that these abilities contribute to general cognitive functioning but do not have a direct link to PM. Language ability is the only variable that consistently correlates with performance on all PM tasks after controlling for age. In our research, we ensured that only those children who understood the instructions and were able to repeat them in their own words were included in the analysis. Therefore, future research should investigate why language skills are important for PM. One hypothesis worth considering is the possibility of tusing children’s own instructions. Furthermore, the inclusion of more than one task to measure language ability is a reasonable approach.

Switching was hypothesized based on theory as a crucial ability necessary for accurately executing a PM task, as children must switch between performing the OT and searching for the prospective cue (see Zuber et al., 2019, for a review). In our study, we found that switching exhibited either very weak or no correlation with PM nonfocal tasks performance. However, due to the meticulous design of the switching task, which is a novel, standardized, valid, and reliable psychological test specifically tailored to assessing switching in children of this age group, it is unlikely that measurement error could account for the lack of correlation with prospective task performance. On the other hand, the absence of correlation between planning and prospective task performance may be attributed to the potential difficulty level of the task. Lavis (2021) conducted research that supports the consideration of an additional explanation. They suggest that in order for cognitive abilities to enhance PM performance, children must first recognize the limitations of their abilities. This enables effective utilization when required in a PM task.

Upon closer examination of the results, it appears that the association between PM and cognitive abilities is weakened among older children. These children generally exhibit higher overall functioning, and the PM task itself did not pose any difficulty for them, even in its most challenging version. In fact, only RM and fluid intelligence hold significance for these eldest children. This implies that individuals who have a good memory for ‘what to do’ may not have difficulty remembering ‘that they have to do it.’ Age appears to be a factor in the accuracy of prospective task execution in younger children. Further research is needed to explore the reasons for this phenomenon. It is possible that factors beyond cognitive abilities, such as task comprehension, cooperation with experimenters, communication skills, familiarity with the testing environment, and acclimatization to one-on-one interactions with unfamiliar adults, may also have an impact. It is important to maintain a balanced and objective approach to the subject matter. Therefore, future studies should focus on including social factors.

The current study has also practical implications. Understanding the factors that contribute to PM has the potential to facilitate and enhance interventions aimed at supporting it. Therefore, it is feasible to implement exercises that target not only PM but also other relevant cognitive abilities. Encouraging the development of PM during preschool attendance is especially important, for example, for children with overprotective parents who do not place demands on their children. In such cases, PM of children with protective parents is lower compared to children with less protective parents (Hajdas et al., 2010). By enhancing both PM and other related cognitive skills, interventions may better prepare children to cope with the increasing demands of performing planned tasks after delay, which increases significantly when formal schooling begins.

Furthermore, it is important to note that our study has several limitations. For future research, particularly when studying a broad age range, it is recommended to increase the difficulty or diversity of the prospective task. Therefore, conclusions drawn from the study should be interpreted with caution. It is important to note in tasks measuring cognitive abilities among 2-year-olds, there were several instances of missing data, resulting in a relatively small group size. Future studies should ensure that all children complete all tasks. Additionally, as previously mentioned, non-cognitive factors appear to significantly impact prospective task performance. Therefore, controlling for sociodemographic variables should be considered in future studies.

## 5 Conclusion

The study investigated the relationship between various cognitive abilities and PM, focusing on potential cognitive mediators of age-related effects on PM performance. The findings support previous indications that even very young children can perform event-based PM tasks, albeit with limited proficiency. The correlation between PM accuracy and age was positive, with significant improvement in performance observed between the ages of 3 and 4. Superior performance was noted for focal tasks compared to nonfocal tasks.

The performance of preschoolers in PM tasks exhibited correlations with multiple cognitive abilities, prominently fluid intelligence and RM, but also inhibitory control, WM, and language ability. The study found that the correlations between age and PM performance varied depending on the child’s age and the task’s nature. Cognitive abilities partially mediated the relationship between age and PM performance for both focal and nonfocal tasks.

In conclusion, this study comprehensively explores the role of age and fundamental cognitive abilities in the PM performance of preschool-aged children. Therefore, the findings provide valuable directions for future longitudinal research.

## Data availability statement

The datasets used and analyzed during the current study are available from the corresponding author on reasonable request. Requests to access these datasets should be directed to ES, elzbieta. szpakiewicz@uken.krakow.pl.

## Ethics statement

The studies involving humans were approved by the Ethics Committee of the Jagiellonian University; approval number: KE/02/112017. The studies were conducted in accordance with the local legislation and institutional requirements. Written informed consent for participation in this study was provided by the participants’ legal guardians/next of kin.

## Author contributions

ES: Writing – original draft, Project administration, Methodology, Investigation, Funding acquisition, Formal analysis, Data curation, Conceptualization. MS-N: Writing – review & editing, Formal analysis.

## Appendix

TABLE 1A Descriptive statistics for cognitive abilities.

**Table:**

	M	SD	Skew.	Kurt.	Min.	Max.
Intelligence	17.80	6.21	0.31	−0.56	5	35.00
Language ability	50.67	22.55	−0.54	−1.09	2	82.00
Attention	36.93	16.92	0.04	−0.77	0	72.00
Retrospective memory	68.41	18.87	−0.39	−1.09	25	97.50
Planning	3.78	2.64	0.18	−1.15	0	9.00
Switching	7.38	2.21	0.61	−0.06	1	12.00
Inhibitory control	6.86	2.56	−0.61	−0.18	0	11.33
Working memory	13.94	10.13	0.79	0.29	0	49.38

In the table, the raw scores are presented.

TABLE 2A Detailed results of the ANOVA analysis.

**Table:**

	Sum of squares type III	df	Mean square	F	*p*	Partial Eta squared	Power^a^
Cue salience	3.14	1	3.14	0.59	0.442	0.003	0.12
Age group	566.16	4	141.54	26.72	<0.001	0.333	1.00
Cue salience * age group	12.19	4	3.05	0.58	0.681	0.011	0.19
Focality	41.29	1	41.29	10.87	0.001	0.048	0.91
Focality * cue salience	5.66	1	5.66	1.49	0.224	0.007	0.23
Focality * Age group	29.79	4	7.45	1.96	0.102	0.035	0.58
Focality * Cue salience * Age	11.39	4	2.85	0.75	0.559	0.014	0.24

^a^Calculated with alfa = 0.05.
